# High Levels of Systemic Inflammatory Markers Associated with Metastasis Incidence in Osteosarcoma

**DOI:** 10.1055/s-0045-1810032

**Published:** 2025-09-08

**Authors:** I. Gede Eka Wiratnaya, Mohamad Dimas Ismail, Risang Haryo Raditya, I. Putu Dema Prasetya

**Affiliations:** 1 Department of Orthopedics and Traumatology, Faculty of Medicine, Udayana University, Prof. Ngoerah General Hospital, Bali, Indonesia

**Keywords:** blood sedimentation, bone neoplasms, C-reactive protein, L-lactate dehydrogenase, osteosarcoma, L-lactato desidrogenase, neoplasias ósseas, osteossarcoma, proteína c-reativa, sedimentação sanguínea

## Abstract

**Objective:**

The present study explores the association between these inflammatory markers and metastasis in osteosarcoma patients.

**Methods:**

We conducted a retrospective analysis of osteosarcoma patients at our center between January 2022 and August 2024. We collected the clinical and laboratory data of the patients, including white blood cell differential count, C-reactive protein (CRP) levels, erythrocyte sedimentation rate (ESR), neutrophil-to-lymphocyte ratio (NLR), alkaline phosphatase (ALP) and lactate dehydrogenase (LDH) levels. Statistical correlation between these markers and metastasis was assessed using the Pearson's Chi-squared test, the Fisher's exact test, and logistic regression.

**Results:**

A total of 40 osteosarcoma patients were included. Elevated levels of neutrophil count ( *p* = 0.024; odds ratio [OR] = 12.667; 95%CI = 1.402–114.419), CRP ( *p* < 0.001; OR = 17.000; 95%CI = 3.464–83.436), ESR ( *p* = 0.009; OR = 19.000; 95%CI = 2.119–170.383), NLR ( *p* = 0.006; OR = 7.429; 95%CI = 1.778–31.040), and LDH ( *p* = 0.009; OR = 9.333; 95%CI = 2.180–39.962) were significantly associated with the presence of metastasis.

**Conclusion:**

The neutrophil count, ESR, NLR, and the levels of CRP and LDH are significantly associated with metastasis in osteosarcoma, and they may serve as valuable prognostic markers. Future research should focus on elucidating the mechanisms underlying this relationship and exploring therapeutic interventions targeting inflammation to mitigate metastasis.

## Resumo

**Objetivo:**

Este estudo explora a associação entre esses marcadores inflamatórios e metástase em pacientes com osteossarcoma.

**Métodos:**

Realizamos uma análise retrospectiva de pacientes com osteossarcoma de nosso centro entre janeiro de 2022 e agosto de 2024. Coletamos dados clínicos e laboratoriais dos pacientes, incluindo contagem diferencial de leucócitos, níveis de proteína C reativa (PCR), velocidade de hemossedimentação (VHS), razão neutrófilo-linfócito (RNL) e concentrações de fosfatase alcalina (FA) e lactato desidrogenase (LDH). A correlação estatística entre esses marcadores e metástase foi avaliada por meio dos teste do Qui-quadrado de Pearson, exato de Fisher e regressão logística.

**Resultados:**

O estudo incluiu 40 pacientes com osteossarcoma. Níveis altos de contagem de neutrófilos ( *p* = 0,024; razão de chances [RC] = 12,667; IC95% = 1,402–114,419) de PCR ( *p* < 0,001; RC = 17,000; IC95% = 3,464–83,436), VHS ( *p* = 0,009; RC = 19,000; IC95% = 2,119–170,383), RNL ( *p* = 0,006; RC = 7,429; IC95% = 1,778–31,040) e LDH ( *p* = 0,009; RC = 9,333; IC95% = 2,180–39,962) foram significativamente associados à presença de metástase.

**Conclusão:**

A contagem de neutrófilos, a VHS, a RNL e os níveis de PCR e LDH estão significativamente associados à metástase em osteossarcoma, e podem ser marcadores prognósticos valiosos. Pesquisas futuras devem se concentrar em elucidar os mecanismos subjacentes a essa relação e explorar intervenções terapêuticas direcionadas à inflamação para mitigar a metástase.

## Introduction

Osteosarcoma is the most common primary malignant bone tumor, and it mainly affects children and young adults. Despite the advancements in treatment protocols incorporating surgery and chemotherapy, osteosarcoma remains associated with a high incidence of metastasis, particularly to the lungs, which contributes significantly to the poor prognosis observed in many patients. 1 Metastasis is the leading cause of mortality in osteosarcoma, with the 5-year survival rate for patients with metastatic disease being lower than 30%, compared with ∼ 70% in patients with localized disease. Understanding the mechanisms underlying metastasis and identifying biomarkers that predict metastatic potential are crucial to improve clinical outcomes. 2

One area that has garnered increasing attention in recent years is the role of inflammation in cancer progression. Inflammation, as part of the body's immune response, has long been implicated in tumor initiation and development, with a growing body of evidence suggesting that chronic inflammation may facilitate tumor metastasis. 3 This relationship is mediated by the tumor microenvironment, which includes various immune cells, cytokines, and other inflammatory mediators that interact with tumor cells to promote proliferation, invasion, and migration. 4

In this context, systemic inflammatory markers, such as C-reactive protein (CRP), erythrocyte sedimentation rate (ESR), neutrophil-to-lymphocyte ratio (NLR), and platelet-to-lymphocyte ratio (PLR), have emerged as potential indicators of cancer prognosis and metastatic potential across a wide range of malignancies. 5 These markers reflect our systemic inflammatory response, which, when dysregulated, may contribute to tumor progression and metastasis. Elevated levels of these markers have been associated with poor clinical outcomes, including increased tumor aggressiveness and metastatic spread in colorectal, breast, and lung cancers, for example. 6 7 8

In osteosarcoma, the role of inflammatory markers in predicting metastasis remains an area of active investigation. Several studies 9 10 11 have suggested that elevated levels of inflammatory marker have been linked to worse survival outcomes, suggesting that these markers could serve as useful prognostic tools in the clinical practice. However, the precise mechanisms through which these inflammatory markers influence osteosarcoma metastasis are not fully understood.

Understanding the relationship between inflammation and metastasis in osteosarcoma could have significant clinical implications. First, identifying reliable inflammatory biomarkers for metastasis could enhance risk stratification, enabling more personalized treatment approaches and earlier interventions for high-risk patients. Furthermore, targeting the inflammatory pathways involved in osteosarcoma progression may offer novel therapeutic strategies to prevent or mitigate metastasis, potentially improving survival outcomes in this aggressive malignancy.

The present study aims to investigate the correlation between inflammatory markers and the incidence of metastasis in osteosarcoma patients. By evaluating the levels of key systemic inflammatory markers, such as CRP, ESR, NLR, and PLR in osteosarcoma patients, we seek to elucidate their potential role as predictive biomarkers for metastatic disease.

## Materials and Methods

### Study Design

The current research employs a retrospective methodology encompassing patients diagnosed with osteosarcoma based on histopathological findings. The individuals diagnosed with osteosarcoma at our facility formed the cohort between the months of January 2022 and August 2024. The independent variables analyzed comprised gender, age, tumor site, and the inflammatory markers (CRP, ESR, alkaline phosphatase [ALP], lactate dehydrogenase [LDH], and NLR), while the dependent variable was the incidence of metastasis. We explored how these various independent factors relate to and affect the likelihood of developing metastasis in individuals diagnosed with osteosarcoma.

### Patient Population

The eligibility criteria for the study were as follows: 1) osteosarcoma was confirmed histologically through biopsy; 2) the patients had undergone a complete blood count (including ESR, NLR, and the levels of CRP, ALP, and LDH) prior to the surgical biopsy; and 3) the patients were identified as having metastasis through radiological examinations. Conversely, we excluded individuals with other comorbid conditions, such as immunodeficiency, infections, and diabetes mellitus. Additionally, patients with low-grade malignancies and those lacking complete blood specimen results for analysis were also omitted. Ethical approval was granted by the ethics committee of our institution under protocol number 2024.02.1.0930. Informed consent was not required because the present study did not involve interventions in patients.

### Data Collection

From the medical records, we systematically recorded demographic and clinical information, including patient gender, age, tumor site, presence of metastasis, site of metastasis, and serum levels of CRP, ESR, NLR, ALP, and LDH. The complete blood count results were retrieved from the patients at the initial visit in our facility. Patients were further categorized based on their metastasis status. To mitigate potential biases, we established clear inclusion and exclusion criteria for the study population. Additionally, the authors responsible for selecting and analyzing the patient data were blinded to the osteosarcoma outcomes. Standardized protocols and instruments were employed at our institution to measure the relevant variables, thereby minimizing information bias. Furthermore, data from hospital medical records were corroborated with direct measurement records from our Orthopedics and Traumatology Department.

### Statistical Analysis

Correlation analysis was conducted using the Pearson's Chi-squared test and the Fisher's exact test to evaluate the association involving clinical and laboratory characteristics and the incidence of metastasis. Clinical and laboratory data were subjected to backward logistic regression analysis to ascertain which features remained significant when evaluated concurrently. The variables incorporated in this analysis had *p* -values < 0.05 from the preceding examination of bivariate correlation.

## Results

### Demographic and Clinical Characteristics of the Patients

A total of 40 patients who met the inclusion criteria were enrolled in the study. All patients were diagnosed with osteosarcoma at our institution between January 2022 and August 2024. Using a total sampling method, all eligible patients were included without random selection. The patients were then divided into two groups: those with metastasis and those without it, based on radiological examination results. The sample consisted of 20 (50%) men and 20 (50%) women. The proportions of the patients in the first, second, third, and fourth or more decades of life were of 3 (7.5%), 21 (52.5%), 5 (12.5%), and 11 (27.5%) respectively. The median age was of 18 (range: 9–82) years. The proportions of tumor locations were of 22 (55%) at the femur, followed by 7 (17.5%) at the tibia, 7 (17.5%) at the humerus, and 4 at other locations: 1 (2.5%) at the fibula, 1 (2.5%) at the radius, 1 (2.5%) at the ulna, and 1 (2.5%) at clavicle. Among the patients with metastasis, 18 (90%) cases were detected in the lung, while, 2 (10%), in the brain ( Table 1 ).

**Table 1:** Clinical characteristics of the patients

Variable	Patients (n = 40)
**Median age (range) in years**	18 (9–82)
1st decade	3 (7.5%)
2nd decade	21 (52.5%)
3rd decade	5 (12.5%)
≥ 4th decade and above	11 (27.5%)
**Gender**	
Male	20 (50%)
Female	20 (50%)
**Site**	
Femur	22 (55%)
Tibia	7 (17.5%)
Fibula	1 (2.5%)
Humerus	7 (17.5%)
Radius	1 (2.5%)
Ulna	1 (2.5%)
Clavicle	1 (2.5%)
**Presence of metastasis**	
Yes	20 (50%)
No	20 (50%)
**Metastasis site**	
Lung	18 (90%)
Brain	2 (10%)

### Correlation between Clinical Laboratory Results and Metastasis

The results of the peripheral blood test demonstrated that neutrophil, monocyte, ALP, LDH, ESR, CRP, and NLR levels in the metastasis group were higher compared with the control group. The laboratory results of the peripheral blood analysis also demonstrated an abnormal distribution in most of the markers (neutrophil, monocyte, eosinophil, basophil, ALP, LDH, CRP, ESR, and NLR) except for the lymphocytes. Furthermore, the data with abnormal distribution was analyzed using the Mann-Whitney test, while the normally-distributed data, using the independent *t* -test. The results indicated that neutrophil ( *p* = 0.01), monocyte ( *p* = 0.033), LDH ( *p* < 0.001), ESR ( *p* < 0.001), CRP ( *p* < 0.001), and NLR ( *p* = 0.012) have a statistically significant correlation with the incidence of metastasis in osteosarcoma patients ( Table 2 ).

**Table 2:** Correlation between inflammation marker and the presence of metastasis

Variable	Metastasis (mean ± standard deviation)	No metastasis (mean ± standard deviation)	*p* -value
**Neutrophil**	8.10 ± 3.94	5.05 ± 1.66	0.010 ^b^
**Lymphocyte**	2.04 ± 0.54	2.04 ± 0.74	0.240 ^a^
**Monocyte**	0.81 ± 0.47	0.52 ± 0.17	0.033 ^b^
**Eosinophil**	0.21 ± 0.24	0.32 ± 0.26	0.091 ^b^
**Basophil**	0.03 ± 0.02	0.04 ± 0.02	0.445 ^b^
**Alkaline phosphatase**	382.95 ± 367.71	180.20 ± 107.46	0.076 ^b^
**Lactate dehydrogenase**	674.25 ± 828.77	240.35 ± 134.15	< 0.001 ^b^
**Erythrocyte sedimentation rate**	67.47 ± 31.95	20.30 ± 1.84	< 0.001 ^b^
**C-reactive protein**	53.89 ± 53.63	4.24 ± 5.30	< 0.001 ^b^
**Neutrophil-to-lymphocyte ratio**	4.20 ± 2.47	2.49 ± 1.67	0.012 ^b^

**Notes:**^a^ independent *t* -test; ^b^ Mann-Whitney test.

The bivariate analysis demonstrated that a high level of several inflammatory markers such as neutrophil ( *p* = 0.008; odds ratio [OR] = 2.296; 95%CI = 1.393–3.784), monocyte ( *p* = 0.035; OR = 2.250; 95%CI = 1.562–3.242), ESR ( *p* = 0.001; OR = 7.207; 95%CI = 1.092–47.577), CRP ( *p* < 0.001; OR = 4.636; 95%CI = 1.610–13.350), NLR ( *p* = 0.004; OR = 2.513; 95%CI = 1.283–4.919), ALP ( *p* = 0.017; OR = 2.333; 95%CI = 1.592–3.421) and LDH ( *p* = 0.001; OR = 3.273; 95%CI = 1.329–8.060) were significantly associated with the incidence of metastasis in osteosarcoma patients ( Table 3 ).

**Table 3:** Bivariate analysis of inflammatory marker and the incidence of metastasis

Variable	Metastasis	No metastasis	*p* -value	Odds ratio (95%CI)
**Neutrophil**			**0.008**	2.296 (1.393–3.784)
Elevated	8 (88.9%)	1 (11.1%)		
Normal	12 (38.7%)	19 (61.3%)		
**Lymphocyte**			−	−
Elevated	0 (0%)	0 (0%)		
Normal	20 (50%)	20 (50%)		
**Monocyte**			**0.035**	2.250 (1.562–3.242)
Elevated	4 (100%)	0 (0%)		
Normal	16 (44.4%)	20 (55.6%)		
**Eosinophil**			0.376	0.630 (0.194–2.039)
Elevated	2 (33.3%)	4 (66.7%)		
Normal	18 (52.9%)	16 (47.1%)		
**Basophil**			−	−
Elevated	0 (0%)	0 (0%)		
Normal	20 (50%)	20 (50%)		
**Alkaline phosphatase**			**0.017**	2.333 (1.592–3.421)
Elevated	5 (100%)	0 (0%)		
Normal	15 (42.9%)	20 (57.1%)		
**Lactate dehydrogenase**			**0.001**	3.273 (1.329–8.060)
Elevated	16 (72.7%)	6 (27.3%)		
Normal	4 (22.2%)	12 (77.8%)		
**Erythrocyte sedimentation rate**			**0.001**	7.207 (1.092–47.577)
Elevated	19 (65.5%)	10 (34.5%)		
Normal	1 (9.1%)	10 (90.9%)		
**C-reactive protein**			**< 0.001**	4.636 (1.610–13.350)
Elevated	17 (77.3%)	5 (22.7%)		
Normal	3 (16.7%)	15 (83.3%)		
**Neutrophil-to-lymphocyte ratio**			**0.004**	2.513 (1.283–4.919)
Elevated	13 (76.5%)	4 (23.5%)		
Normal	7 (30.4%)	16 (69.6%)		

The multivariate analysis demonstrated that among the elevated inflammatory markers available, high levels of neutrophil count ( *p* = 0.024; OR = 12.667; 95%CI = 1.402–114.419), ESR ( *p* = 0.009; OR = 19.000; 95%CI = 2.119–170.383), CRP ( *p* < 0.001; OR = 17.000; 95%CI = 3.464–83.436), NLR ( *p* = 0.006; OR = 7.429; 95%CI = 1.778–31.040), and LDH ( *p* = 0.009; OR = 9.333; 95%CI = 2.180–39.962) were associated with the incidence of metastasis in osteosarcoma patients ( Table 4 ).

**Table 4:** Multivariate analysis of inflammatory markers and incidence of metastasis

Inflammatory markers	Value	Standard error	95%CI	Odds ratio	*p* -value
**Neutrophil**	2.539	1.123	1.402–114.419	12.667	**0.024**
**Lactate dehydrogenase**	2.234	0.742	2.180–39.962	9.333	**0.003**
**Erythrocyte sedimentation rate**	2.944	1.119	2.119–170.383	19.000	**0.009**
**C-reactive protein**	2.833	0.812	3.464–83.436	17.000	**< 0.001**
**Neutrophil-to-lymphocyte ratio**	2.005	0.730	1.778–31.040	7.429	**0.006**

## Discussion

The results of the current study indicate that elevated neutrophil count, ESR, NLR, as well as elevated levels of CRP and LDH are significantly associated with the presence of metastasis in osteosarcoma patients. These results align with those of the existing literature that supports the role of systemic inflammation in cancer progression, particularly in promoting metastasis.

### Inflammation as a Driver of Metastasis

Chronic inflammation has long been recognized as a hallmark of cancer progression. 12 In the context of osteosarcoma, inflammation within the tumor microenvironment creates a favorable environment for tumor cells to evade immune surveillance, promote angiogenesis, and facilitate invasion and metastasis. 13 14 Tumor-associated inflammation is not simply a side effect; it actively contributes to the cancer's aggressiveness through various pathways. Pro-inflammatory cytokines, chemokines, and immune cells are integral components of this process, orchestrating an environment that supports the growth, survival, and dissemination of tumor cells. 15

In osteosarcoma, metastasis, particularly to the lungs, is a critical determinant of prognosis. Metastatic osteosarcoma is characterized by more aggressive tumor behavior, often associated with a higher degree of systemic inflammation. 1 16 The markers identified in the present study (CRP, ESR, NLR, and LDH) reflect distinct yet interconnected aspects of this inflammatory response, providing insight into the mechanisms by which inflammation may drive metastatic progression.

### C-Reactive Protein and Metastasis

C-reactive protein is an acute-phase protein synthesized in the liver in response to pro-inflammatory cytokines, primarily interleukin-6 (IL-6) and tumor necrosis factor-alpha (TNF-α). 17 Elevated CRP levels have been consistently associated with poor prognosis and increased metastatic potential in various cancers, including osteosarcoma. 6 18 19 20 The significant association between elevated CRP levels and the presence of metastasis in the current study suggests that CRP may serve as a surrogate marker for the extent of systemic inflammation and tumor aggressiveness in osteosarcoma.

By activating the complement system and promoting the recruitment of immune cells to the tumor microenvironment, CRP plays a role in amplifying the inflammatory response. This heightened inflammatory state facilitates tumor progression through several mechanisms. First, chronic inflammation can lead to increased vascular permeability and angiogenesis, both of which are critical for tumor cells to invade surrounding tissues and enter the bloodstream. Second, the inflammatory cytokines that drive CRP production, such as IL-6 and TNF-α, have been shown 4 5 to promote epithelial-mesenchymal transition (EMT), a process through which cancer cells acquire migratory and invasive properties necessary for metastasis.

Additionally, CRP has been implicated in the suppression of antitumor immunity. High levels of CRP may inhibit the activity of cytotoxic T-cells and natural killer (NK) cells, which are essential for recognizing and eliminating metastatic cancer cells. 21 This immune evasion enables tumor cells to disseminate more freely and cause metastatic lesions in distant organs.

### Erythrocyte Sedimentation Rate and Tumor Aggressiveness

The ESR is another marker of systemic inflammation that reflects the degree of red blood cell aggregation, which is influenced by the presence of acute-phase proteins such as fibrinogen and CRP. An elevated ESR is indicative of an ongoing inflammatory process, and it has been associated with poor outcomes in several cancers. 7 22 In osteosarcoma, the present study demonstrates a significant correlation between high ESR and the presence of metastasis, suggesting that increased systemic inflammation may contribute to the aggressive behavior of metastatic osteosarcoma.

The association between high ESR and metastasis may be related to the chronic inflammatory environment that promotes tumor progression. Similar to CRP, elevated ESR is likely driven by proinflammatory cytokines such as IL-6 and TNF-α, which are known to play a role in osteosarcoma pathogenesis. These cytokines can enhance the invasive capacity of tumor cells by promoting the breakdown of the extracellular matrix and facilitating the degradation of tissue barriers, thereby enabling tumor cells to migrate and establish metastatic sites. 23

### Neutrophil-to-Lymphocyte Ratio as a Prognostic Indicator

The NLR is a widely studied marker of the systemic inflammatory response, representing the balance between protumor neutrophils and antitumor lymphocytes. An elevated NLR, as observed in the current study, has been correlated with poor prognosis and increased metastatic potential in a variety of malignancies, including osteosarcoma. The significant association between high NLR and metastasis in osteosarcoma highlights the role of neutrophils in promoting tumor growth and of lymphocytes in defending against tumor progression. 24

Neutrophils contribute to the metastatic process through several mechanisms. Tumor-associated neutrophils (TANs) can promote angiogenesis by secreting vascular endothelial growth factor (VEGF), enhancing the tumor's blood supply and facilitating metastatic spread. They also release proteolytic enzymes, such as matrix metalloproteinases (MMPs), which degrade the extracellular matrix and promote tumor cell invasion. Furthermore, neutrophils can suppress adaptive immune responses by inhibiting the activity of cytotoxic T-cells and NK cells, further facilitating immune evasion and metastasis. 25

On the other hand, lymphocytes, particularly T-cells, play a crucial role in antitumor immunity. A low lymphocyte count, as reflected by a high NLR, indicates impaired immune surveillance and a reduced ability to control tumor growth and metastasis. Thus, a high NLR suggests an imbalance between protumor and antitumor forces, favoring metastatic dissemination. 25

### Lactate Dehydrogenase and Tumor Metabolism

Lactate dehydrogenase is an enzyme involved in anaerobic glycolysis and is often elevated in rapidly-proliferating tumor cells. 26 High LDH levels have been associated 2 with aggressive tumor biology, treatment resistance, and poor outcomes in osteosarcoma. In the present study, elevated LDH levels were significantly correlated with the presence of metastasis, underscoring its potential role as a marker of tumor aggressiveness and metastatic potential.

The link between LDH and metastasis can be understood in the context of the Warburg effect, a metabolic adaptation in which cancer cells preferentially use glycolysis for energy production, even in the presence of oxygen. This shift toward glycolysis leads to increased lactate production, which acidifies the tumor microenvironment and promotes tumor invasion and metastasis. High LDH levels reflect this altered metabolism and are often associated with increased tumor cell proliferation and metastatic capability. 27

Moreover, lactate itself can act as a signaling molecule within the tumor microenvironment, promoting angiogenesis, immune evasion, and tissue remodeling, all processes that facilitate metastasis. Elevated LDH levels also reflect the hypoxic conditions within the tumor, which are known to drive the selection of more aggressive, metastatic cell clones. 28

Despite the statistically significant association observed between the inflammatory markers and metastasis, the current study have several limitations, such as the retrospective design, which relies on preexisting medical records that may vary in quality and completeness. This reliance can introduce selection bias, as only patients meeting the specific inclusion criteria with available, complete data were included, potentially excluding cases that might influence the results. Additionally, the retrospective nature limits control over the confounding factors, such as variations in diagnostic techniques or treatment protocols, which could impact the study outcomes. These limitations may affect the generalizability of the findings to broader populations, underscoring the need for prospective studies to validate these results and clarify the causative mechanisms.

## Conclusion

The present study demonstrates that elevated ESR, NLR, as well as elevated levels of CRP, neutrophils, and LDH are significantly associated with metastasis in osteosarcoma patients, highlighting the prognostic value of systemic inflammatory markers and their potential role in guiding risk stratification and personalized treatment strategies.
